# Beyond “you are what you eat”: Unlocking gut microbiota‐mediated biotransformation of dietary phytochemicals

**DOI:** 10.1002/imo2.70097

**Published:** 2026-04-18

**Authors:** Peng Yang, Ren‐You Gan

**Affiliations:** ^1^ Department of Food Science and Nutrition The Hong Kong Polytechnic University, Hung Hom Kowloon Hong Kong SAR China; ^2^ Research Institute for Future Food The Hong Kong Polytechnic University, Hung Hom Kowloon Hong Kong SAR China

## Abstract

The “efficacy paradox” of phytochemicals, low bioavailability yet significant health benefits, is associated with gut microbiota, which biotransforms dietary precursors into bioactive metabolites, enabling systemic effects. Thus, health outcomes of diet depend not just on intake “the rainbow”, but on gut microbial metabolism, redefining “you are what you eat” to “you are what your microbes make of what you eat”.

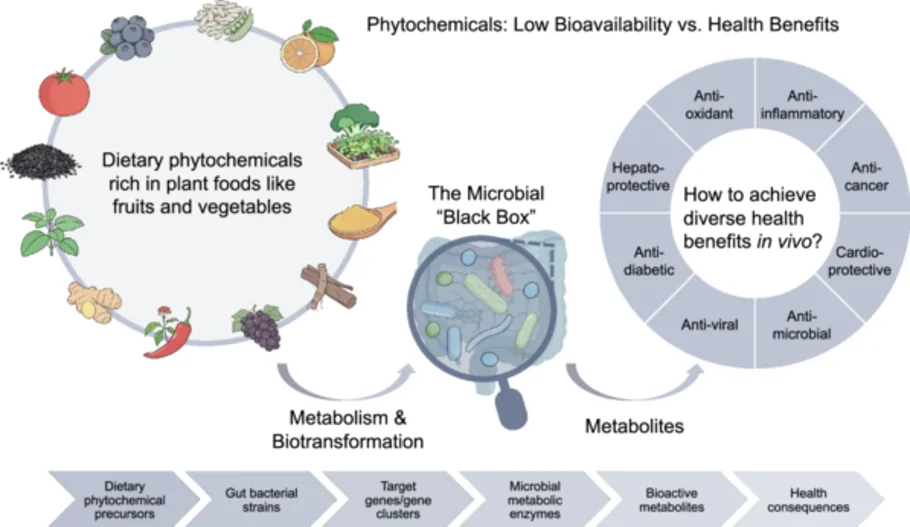

The health‐promoting properties of dietary phytochemicals have been extensively documented in preclinical data from in vitro and animal studies, where they exhibit potent bioactivities [1]. However, when translated into human interventions, the benefits of these compounds are often inconsistent, highly variable across individuals, and sometimes disappointingly ineffective [2, 3, 4]. This disconnect is often termed the “efficacy paradox”, representing a significant bottleneck in the field of nutrition, since it challenges the conventional dietary recommendations and highlights our incomplete understanding of how phytochemicals are metabolized within the human body (Figure 1).

**Figure 1:**
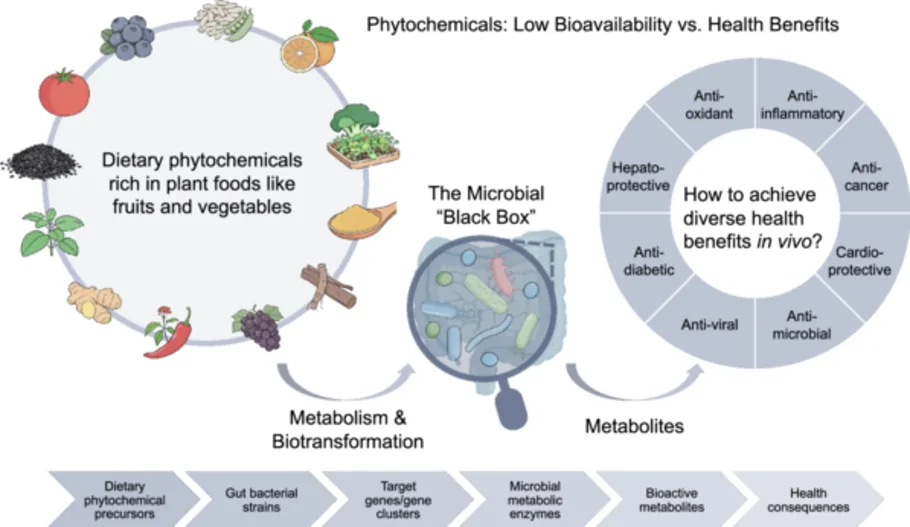
Unlocking the efficacy paradox of phytochemicals from “you are what you eat” to “you are what your microbiota metabolizes”.

Multiple factors contribute to this paradox, including the food matrix, processing methods, dosage, host genetics, hepatic metabolism, and lifestyle. However, a rapidly growing number of evidence indicates that the gut microbiota is the most influential and personalized determinant of phytochemical's efficacy [5]. In fact, the vast majority of dietary phytochemicals (up to 90%–95%) cannot be absorbed in the upper gastrointestinal tract and enter the colon, where the microbial “black box”—trillions of anaerobic microbes, act as an essential, highly personalized, and biological processing unit for phytochemical biotransformation (Figure 2A). These microbes possess a vast and diverse arsenal of metabolic enzymes (e.g., β‐glycosidase, carbohydrate‐active enzymes (CAZymes), esterase, reductase, decarboxylase, dehydroxylase, demethylases, and dehydrogenases) that are most absent in the human host, enabling them to deconstruct the complex dietary precursors into smaller, more lipophilic, and often more bioactive metabolites that can be readily absorbed through the colonic epithelium into circulation and exert systemic health effect [6]. Consequently, the composition of individual's gut microbiota determines whether a specific phytochemical is activated into its potent form, giving rise to the concept of “metabotypes” (metabolic phenotypes), the distinct population subgroups defined by their ability to produce certain key metabolites [7].

**Figure 2:**
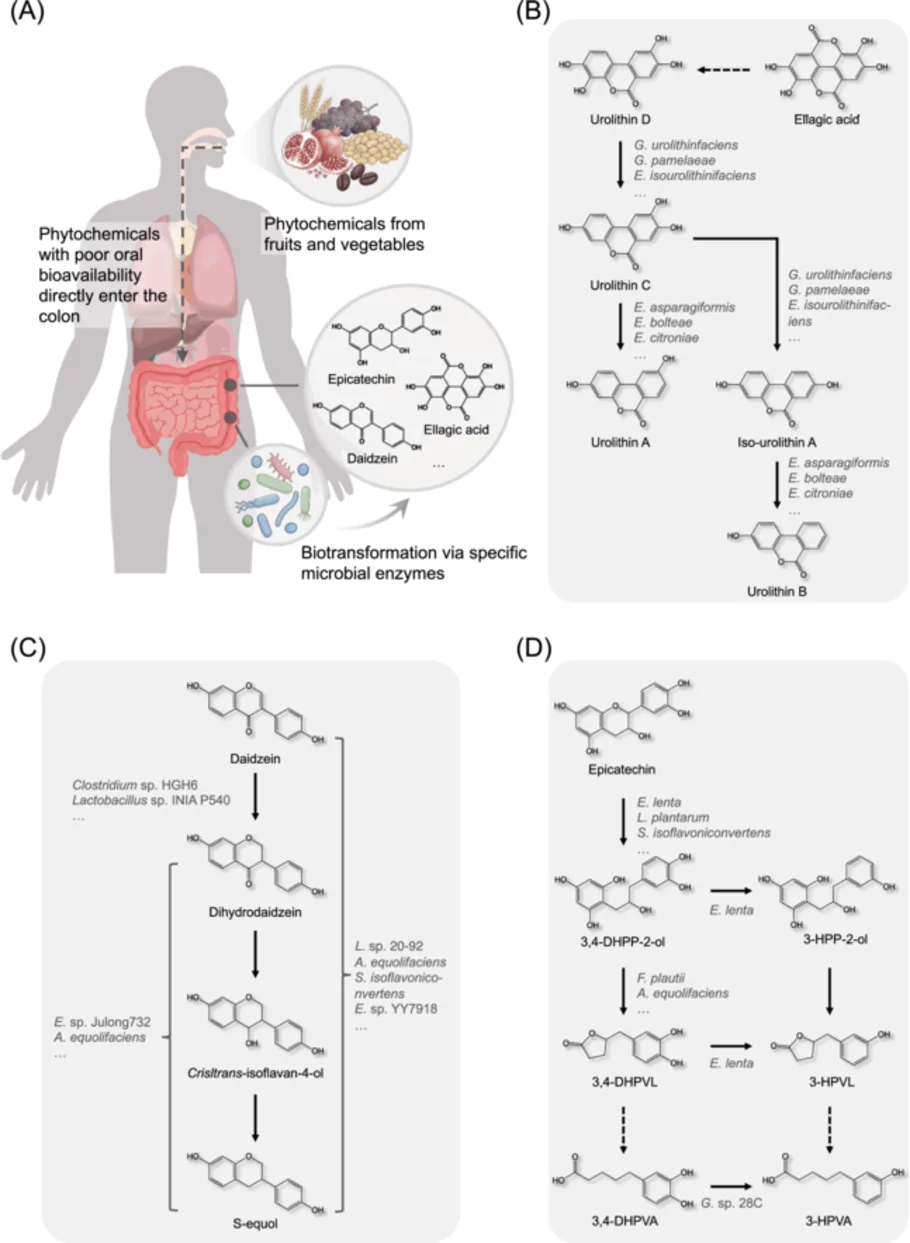
Biotransformation pathways of phytochemicals. (A) The fate of diets rich in phytochemicals. (B) Ellagitannins to urolithins. (C) Daidzein to S‐equol. (D) Epicatechin to PVLs. DXN, α,β‐dihydro‐xanthohumol; DDXN, desmethyl‐α,β‐dihydro‐xanthohumol; 3,4‐DHPP‐2‐ol, 1‐(3′,4′‐dihydroxyphenyl)−3‐(2″,4″,6″‐trihydroxyphenyl)‐propan‐2‐ol; 3‐HPP‐2‐ol, 1‐(3′‐hydroxyphenyl)−3‐(2″,4″,6″‐trihydroxyphenyl)‐propan‐2‐ol; 3,4‐DHPVL, 5‐(3′,4′‐dihydroxyphenyl)‐γ‐valerolactone; 3‐HPVL, 5‐(3′‐hydroxyphenyl)‐γ‐valerolactone; 3,4‐DHPVA, 3‐(3′,4′‐dihydroxyphenyl)valeric acid; 3‐HPVA, 5‐(3′‐Hydroxyphenyl)valeric acid.

Although previous studies have comprehensively identified the biotransformation pathways of specific phytochemicals (e.g., ellagitannins to urolithins, daidzein to S‐equol, and flavan‐3‐ols to phenyl‐γ‐valerolactones), the field now faces a more profound challenge—how can we translate preclinical mechanisms into personalized nutritional interventions? Despite decades of research, several critical questions remain unanswered, like (1) which steps within these pathways are supported by the microbe–enzyme–gene evidence in the human body, rather than inferences from preclinical studies; (2) what limitations exist in the current human research; (3) what is the optimal way to combine multi‐omics and traditional biochemical methods to understand how correlation and causation are connected; (4) and most importantly, how can this mechanistic understanding be translated into personalized nutrition practice?

This work, therefore, aims to discuss the aforementioned questions through a comprehensive framework that closely links mechanistic discoveries with practical applications. Specifically, we rigorously evaluate evidence regarding the biotransformation pathways of key phytochemicals, clearly distinguishing among definitive validation, in vitro inferences, and human trial evidence, while highlighting their respective limitations. Furthermore, we integrate current methodologies into a systematic discovery‐to‐validation strategy encompassing multi‐omics, bioinformatics, and classical biochemistry. Finally, a concrete roadmap for developing next‐generation probiotics (NGPs), precision synbiotics, and postbiotic nutraceuticals is proposed. By doing so, we can unlock the true potential of dietary phytochemicals and pioneer a new generation of truly effective functional ingredients from their gut metabolites.

## 1 UNLOCKING THE BLACK BOX: GUT MICROBIOTA‐MEDIATED METABOLISM PROCESS

The journey to uncover these microbial secrets has yielded fascinating insights, transforming our understanding of how diet influences nutrition and health. In recent years, increasing work has been carried out to answer two fundamental questions involved in gut microbiota‐mediated metabolism of phytochemicals. First, what are the key gut bioactive metabolites of phytochemicals, and how do the key metabolites contribute to the health benefits of phytochemical precursors? Second, how is gut microbiota‐mediated metabolism and biotransformation process implemented, especially specific gut bacteria species/strains, their metabolic enzymes and encoding genes? Several well‐characterized examples are elaborated and discussed below.

### Ellagitannins to urolithins: Emerging star nutraceuticals

Ellagitannins, abundant in many plant‐based foods like pomegranates, berries, and walnuts, are large polyphenols with poor bioavailability. Upon reaching the colon, they are hydrolyzed to ellagic acid (EA) by gastric acid, which is then sequentially converted by gut bacteria into a series of smaller, more absorbable compounds dibenzopyran‐6‐one derivatives known as urolithins (Figure 2B) [8]. The biological significance of this transformation is well‐established. Urolithin A and Urolithin B are the major end‐products of EA, and Urolithin A in particular exhibits significant health functions including antioxidant, anti‐inflammatory, anti‐cancer, anti‐aging, cardioprotective properties, and muscle function‐enhancing effects that exceed those of EA itself [8, 9]. However, this microbial biotransformation is not universal. Based on the gut microbial capacity, individuals can be classified into distinct “urolithin metabotypes”, including metabotype A (produces only Urolithin A), metabotype B (produces Urolithin A, Isourolithin A, and/or Urolithin B), and metabotype 0 (produces no above urolithins at all) [10].

These urolithin metabotypes are directly linked to the presence of a functional microbial “assembly line” with key dehydroxylation capabilities. Foundational work identified *Gordonibacter urolithinfaciens* DSM 27213^T^, and *G. pamelaeae* DSM 19378T capable of converting EA into intermediate urolithins such as Urolithin C [11]. Subsequently, *Ellagibacter isourolithinifaciens* DSM 104140^T^ was isolated and shown to produce isourolithin A from EA [12]. However, these species alone cannot complete the pathway to Urolithin A/B, as they lack the capacity for the final dehydroxylation steps. A subsequent study revealed that certain *Enterocloster* species (e.g., *E. asparagiformis*, *E. bolteae*, and *E. citroniae*) are responsible for this final dehydroxylation step that produce the end‐stage Urolithins A/B, defining each metabotype [13].

The dehydroxylation mechanism, especially the challenging sequential dihydroxylation of urolithins, has been clarified recently at the enzymatic and genetic levels. Pidgeon et al. uncovered an inducible Urolithin C dehydroxylase (*ucd*) operon in *Enterocloster* species, which includes *ucdC* (FAD‐binding subunit), *ucdF* (2Fe2S‐binding subunit), and *ucdO* (molybdopterin cytosine dinucleotide (MCD)‐binding subunit), and this operon encodes a novel molybdopterin‐dependent enzyme complex that specifically dehydroxylates urolithins at its 9‐hydroxy position [14]. To move forward, Bae et al. systematically identified four urolithin dehydroxylases from *Gordonibacter* strains and *E. isourolithinifaciens*, including three catechol dehydroxylases (Eadh1, Eadh2, and Eadh3) belonging to the dimethyl sulfoxide reductase family, and a xanthine oxidase family enzyme (Ucdh) from *E. bolteae* [15]. These enzymes target individual hydroxyl groups on the urolithins and exhibit substrate specificity. For example, Eadh1, Eadh2, and Eadh3 are specifically responsible for dehydroxylating the hydroxy groups at positions 4, 10, and 8 of urolithins, respectively. Therefore, it explains the previous finding that *Gordonibacter* (containing Eadh1/2) can only produce Urolithin C, while *Ellagibacter* (containing Eadh1/2/3) additionally generates Isourolithin A [11, 12, 13]. On the other hand, Ucdh specifically targets on the 9‐position hydroxy group of Urolithin C, which enables *Enterocloster* (containing Ucdh) to produce Urolithin A [14].

Besides, several other gut bacterial strains have also been reported to biotransform ellagitannins or EA, like *Bifidobacterium pseudocatenulatum* INIA P815, *Lactococcus garvieae* FUA009, *Enterococcus faecium* FUA027, *Streptococcus thermophilus* FUA329, and *Lactiplantibacillus plantarum* strains. However, the metabolism efficiency of *Bifidobacterium* and *Lactobacillus* strains is very limited, and their biotransformation mechanism remains uncharacterized. Thus, while they demonstrate that urolithin‐producing capacity may be more widespread than previously appreciated, the evidence for these strains is currently limited to in vitro incubation, therefore, whether they contribute to urolithin production in the complex gut ecosystem of humans is unknown. The observation that *B. pseudocatenulatum* INIA P815 produces Urolithin A and Urolithin B in the BHI medium but not in the RCM medium highlights the critical importance of culture conditions and may explain the difficulty of isolating urolithin‐producing strains from fecal samples [16].

The concept of urolithin metabotypes is strongly supported by observational studies in human cohorts, with consistent classification into metabotype A, B, or 0 regardless of dietary source, age, and health status [10]. Intervention studies with pomegranate extracts have shown that metabotype distribution is stable over time in most individuals, although some metabotype 0 individuals can be converted to producers after a long‐term or high‐dose exposure [8, 16]. Importantly, metabotypes have been associated with differences in gut microbial composition, with Urolithin A producers showing enrichment of *Gordonibacter* species and distinct enterotype profiles [8]. Urolithin A has already entered the clinical trial phase, with trial results indicating its ability to improve mitochondrial health and muscle strength in the elderly [17]. Nevertheless, critical gaps remain that most human studies linking metabotypes to health outcomes are cross‑sectional and underpowered (typically *n* < 100), no large‑scale intervention trial has stratified participants by metabotypes to test whether the baseline metabolic capacity modifies the clinical response to Urolithin A or ellagitannin‐rich foods, the regulation of the *ucd* operon and dehydroxylase expression by dietary factors or host signals has not been explored, and the potential contribution of uncultured microbes to urolithin production remains unaddressed.

### Soy isoflavones to S‐equol: The key to soy's efficacy

Soy isoflavones, particularly daidzein, are well‐known phytoestrogens. Their health benefits, such as alleviating menopausal symptoms, antioxidant, and antiproliferative effects, have been extensively reported but remain controversial because of the interindividual variability [4, 16]. The discovery of S‐equol, a key gut microbial metabolite of daidzein, provides a crucial explanation for this variability in the efficacy of soy isoflavones [4]. In fact, S‐equol exhibits greater biological activity than its precursor, as evidenced by its higher binding affinity to estrogen receptors and stronger antioxidant activity, making it the primary determinant of soy's health effects [3]. However, the capacity of S‐equol production is not universal, since only 20%–30% of adults in Western countries and 50%–70% in many Asian countries are “S‐equol producers” and the remainders are “S‐equol non‐producers”, a trait entirely dependent on harboring the specific gut bacteria [18].

Landmark studies have identified the specific “specialist converters” in S‐equol producers responsible for biotransformation of soy isoflavones, most of which belong to the Eggerthellaceae family, such as *Adlercreutzia equolifaciens*, *Slackia isoflavoniconvertens*, and *Eggerthella sp*. YY7918 [19]. The genetic basis for this conversion has been comprehensively elucidated across multiple producers. Related metabolic enzymes and their encoding gene clusters for S‐equol production, including daidzein reductase (DZNR), dihydrodaidzein reductase (DHDR), and tetrahydrodaidzein reductase (THDR), along with a dihydrodaidzein racemase (DDRC), have been identified in *Slackia sp*. NATTS, *S. isoflavoniconvertens*, *A. equolifaciens* DSM19450^T^, *Eggerthella* sp. YY7918, and other S‐equol‐producing bacteria like *Lactococcus* sp. 20–92. Among these, DZNR is responsible for the initial step to generate dihydrodaidzein. DDRC then converts dihydrodaidzein enantiomer into its isomer, which can be reduced to *cis/trans*‐isoflavan‐4‐ol (tetrahydrodaidzein) by DHDR. Finally, THDR completes the pathway by converting tetrahydrodaidzein into the end product S‐equol (Figure 2C). These findings constitute definitive microbe–enzyme–gene evidence, as they have been validated through heterologous expression in non‐producing hosts (e.g., *Escherichia coli*), gene knockout experiments, and biochemical characterization of recombinant enzymes.

Apart from the strains of the Eggerthellaceae family, other gut microbes in the human, including the strains from *Bifidobacteria* (e.g., *Bifidobacterium breve* 15700 and *B. longum* BB536), *Lactobacillus* (e.g., *L. intestinalis* LC096206.1), and *Lactococcus* (e.g., *L. garvieae* 20–92) genera, have also been reported to generate S‐equol *in vitro*. However, S‐equol‐producing gene clusters were not found in these bacteria based on a taxonomic distribution analysis in representative prokaryotic genomes of 47,894 species [19]. The absence of homologous genes in these taxa indicates that the bioconversion of daidzein to S‐equol is accomplished by distinct enzyme clusters and underscores the necessity for further sequencing and experimental work to understand S‐equol production in these strains.

This microbial distinction between S‐equol producers and non‐producers is one of the best‐established examples of a gut microbiota‐determined metabotype. Numerous observational studies and small‐scale intervention trials have consistently shown that the health benefits of soy isoflavones, particularly for hormone‐dependent diseases, are more pronounced in S‐equol producers [3, 4, 18]. However, the human evidence is still limited. Most studies relied on a single spot urine or plasma measurement to classify producer status, but the persistence of this metabotype within individuals during their lifespan is not well clarified. In addition, most trials did not prospectively stratify participants according to producer status, making it difficult to draw causal conclusions regarding the regulatory role of this metabotype. Besides, the threshold for S‐equol production required to achieve clinical efficacy remains undetermined, and the presence of this gene cluster is variable even among known species harboring it (such as *A. equolifaciens*), indicating that species identification via 16S rRNA sequencing does not guarantee functional capability. This underscores the necessity of employing functional gene‐based approaches [19].

### Flavan‐3‐ols to phenyl‐γ‐valerolactones: Unlocking tea and cocoa's benefits

Flavan‐3‐ols, including monomers such as epicatechin (EC), epigallocatechin (EGC), and their gallate derivatives like epicatechin gallate (ECG) and epigallocatechin gallate (EGCG), as well as polymeric proanthocyanidins, are the most abundant flavonoids in the human diet, with tea, cocoa, apples, and grapes as the major dietary sources. Although a small fraction of monomers can be absorbed in the small intestine, the majority, along with all of their larger polymers, mainly reach the colon [13]. Here, they are catabolized by microbes into a class of signature intermediate metabolites known as phenyl‐γ‐valerolactones (PVLs) and their related phenylvaleric acids (Figure 2D). Specifically, the microbial bioconversion of flavan‐3‐ols undergoes a sequential multi‐step pathway, including the degalloylation of galloylated catechins (e.g., EGCG and ECG) by microbial esterases/tannases to generate gallic acid, which is further decarboxylated to pyrogallol, the C‐ring cleavage of non‐galloylated catechins like EC and EGC to yield diphenylpropan‐2‐ols, the A‐ring fission and lactonization to produce PVLs, and further degradation to smaller phenolic acids. These metabolites, detected as the primary forms in the gut and circulation, are central to mediating the health benefits of dietary flavan‐3‐ols (e.g., EGCG and procyanidins), due to their potent bioactivities like antioxidant, anti‐inflammatory, anti‐cancer, anti‐diabetic, and hypotensive effects [13].

Early studies identified diverse gut bacteria capable of metabolizing flavan‐3‐ols in vitro. For degalloylation, *Enterobacter aerogenes*, *Raoultella planticola*, *Klebsiella pneumoniae*, *Bifidobacterium longum* subsp. infantis, and *Akkermansia muciniphila* have been shown to hydrolyze EGCG to EGC and gallic acid [20, 21]. Lactic acid bacteria such as *L. plantarum* and *Leuconostoc mesenteroides* also possess tannase activity capable of degalloylation [18, 21]. For C‐ring cleavage, several bacterial species belonging to *Actinobacteria phylum* (Eggerthellaceae family) have been identified, including *Eggerthella lenta* JCM 9979, *Adlercreutzia equolifaciens* MT4s‐5, *Slackia equolifaciens* JCM 16059, *Asaccharobacter celatus* JCM 14811, *Eubacterium* sp. SDG‐2, and *Eggerthella lenta* CAT‐1, and these bacteria can reductively cleave the C‐ring of EC and EGC to produce diphenylpropan‐2‐ols such as 3,4‐DHPP‐2‐ol [21]. For the critical step of A‐ring fission, *Flavonifractor plautii* strains (e.g., ak2, ATCC 29863, ATCC 49531, and MT42), belonging to Firmicutes, have been reported to cleave the A‐ring of diphenylpropan‐2‐ols and catalyze lactonization to produce PVLs [20, 21]. Importantly, *F. plautii* appears to be the only genus reported to perform this conversion, and it acts on intermediates derived from both EC and EGC regardless of the B‐ring hydroxylation patterns [21].

Recently, a major advanced study revealed another PVL‐producing strain named *Eggerthella lenta* DSM 2243. This strain can first cleave the C–O bond in the C‐ring of catechin and EC using a novel enzyme, catechin benzyl ether reductase (Cber). Subsequently, regiospecific dehydroxylation of the B‐ring is performed by two distinct enzymes, including catechin dehydroxylase (Cadh) for catechin‐derived intermediates and EC dehydroxylase (eCadh) for intermediates derived from EC [22]. Despite this progress, the enzyme(s) responsible for A‐ring fission remain completely unidentified. *F. plautii* clearly performs this reaction, but the genes and enzymes involved have not been characterized. Neither *E. lenta* nor *F. plautii* alone can complete the full pathway to produce PVLs in the pure culture, suggesting that a consortium of bacteria, or as‐yet‐uncultured microbes, should be required.

Importantly, the gut microbial capacity to produce PVLs varies significantly between individuals, giving rise to distinct “flavan‐3‐ol metabotypes” that may underlie interindividual differences in response to flavan‐3‐ol‐rich foods [20, 22]. Several studies have documented substantial interindividual variation in the PVL production following flavan‐3‐ol consumption. Lessard‐Lord et al. characterized the microbial metabolism of EC in fecal samples from healthy adults and identified three distinct metabotypes based on PVL production profiles [23]. However, there are still some limitations that need to be addressed. For instance, most studies were cross‐sectional studies and had a small number of participants (typically *n* < 50), lacking longitudinal assessment of metabotype stability, and no large‐scale study prospectively stratified participants by metabotypes to test whether baseline PVL‐producing capacity modifies the clinical response to flavan‐3‐ol‐rich foods. In addition, the relationship between in vitro fecal production capacity and actual in vivo systemic exposure (plasma PVL concentrations) remains poorly characterized, and it is largely unknown about the concentration of PVL required to exert biological activities within the target organs.

### Other examples

Besides the above typical examples, many other phytochemicals have been demonstrated to be metabolized and activated by gut microbiota to exert their health functions, such as “isoxanthohumol to 8‐prenylnaringenin” by *Eubacterium limosum* [24], “broccoli glucosinolate to isothiocyanate” by *Bacteroides thetaiotaomicron* [25]. However, gut microbiota‐mediated metabolism and biotransformation mechanisms of many of them remain unclear and are yet to be clarified in the future.

On the other hand, gut microbiota may also inactivate certain phytochemicals since their ability to cleave important functional groups cannot be effectively controlled. Bae et al. found that *Gordonibacter pamelaeae* can dehydroxylate hydrocaffeic acid, an anti‐inflammatory phenolic acid, into an inactive metabolite by its catechol dehydroxylase Hcdh [26]. This example is aligned with two previous classical drug metabolism cases. Parkinson's disease (PD) drug l‐dopa can be inactivated by gut bacteria *Enterococcus faecalis* and *E. lenta*‐mediated biotransformation, producing PD‐inactive dopamine and m‐tyramine, respectively [27], and the cardiac drug digoxin can be reduced and inactivated by gut bacteria *E. lenta* [28]. Therefore, it is complex for the health consequences of gut microbiota‐mediated metabolism.

## 2 THE MODERN TOOLKIT: DECIPHERING HOST–MICROBE–DIET INTERACTIONS

To systematically map these metabolic pathways and move from correlation to causation, nutritional science has adopted a powerful toolkit that integrates multi‐omics and bioinformatics with classic biochemical techniques (Figure 3).

**Figure 3:**
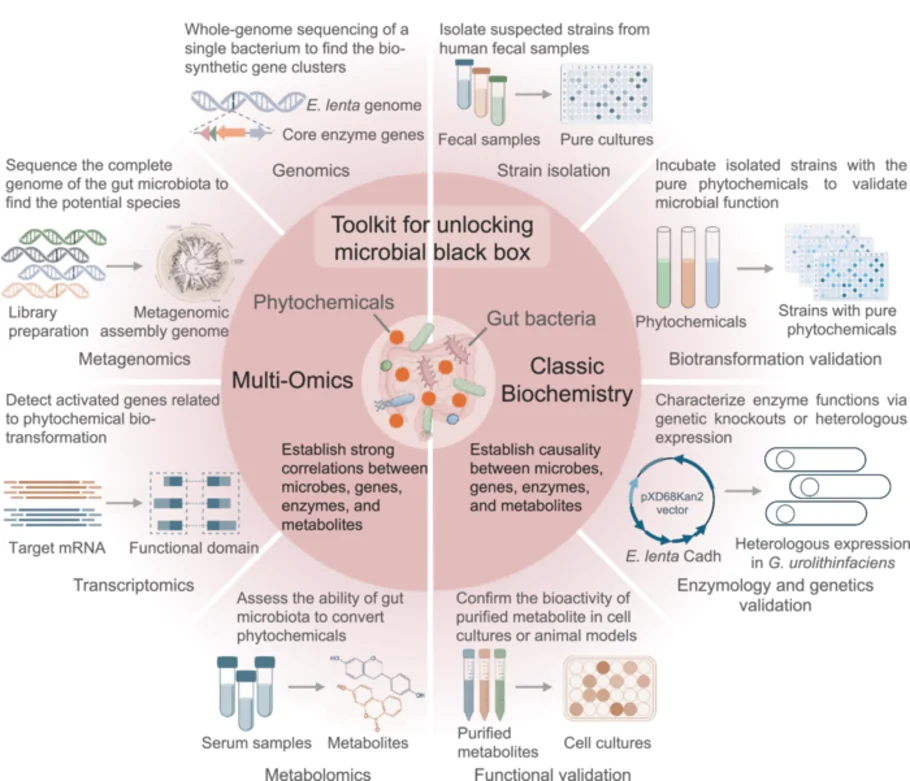
The modern toolkit for unlocking the gut's black box.

### The power of multi‐omics and bioinformatics for targeted gut microbiota mining

The identification of a specific microbial function for phytochemical biotransformation is the first essential step to understand interindividual variability. Strategies relying on traditional methods, although highly effective for isolating functional strains, are inherently low‐throughput and fail to encompass the vast majority of gut microbial diversity [16]. The development of multi‐omics technologies has significantly enhanced our capacity for systematically exploring the metabolic potential of gut microbiota, enabling us to transition from incidental discoveries to targeted, hypothesis‐driven mining of microbial functions [6]. This section integrates current multi‐omics and bioinformatic strategies into a discovery‐to‐validation pipeline, clearly articulating how these tools can be combined to transition from correlation to causation.

The discovery stage aims to connect phytochemical inputs with microbial metabolite outputs and probably host responses. Metagenomics, metabolomics, and transcriptomics can effectively monitor changes in the gut microbiota and its metabolic products under different dietary interventions. This facilitates the rapid identification of potential microorganisms and their associated gene clusters capable of biotransforming phytochemicals.

Metagenomics provides a genetic census of the microbial community, answering “who is there” and “what is their potential function”. Shotgun metagenomic sequencing of fecal DNA can be used to reconstruct microbial genomes and identify genes that code for particular metabolic enzymes. A landmark study by Dufault‐Thompson et al. utilized metagenomic data from 47,894 bacterial genomes to systematically map the taxonomic distribution of S‐equol biosynthesis gene clusters. This revealed their exclusive presence within the Eggerthellaceae family and identified novel species potentially capable of producing S‐equol [19].

Metabolomics is a functional analytical technique employed to examine small molecules present in feces, plasma, and urine, with the objective of identifying microbial metabolites that emerge following the intake of phytochemicals, answering “what they are making”. Untargeted metabolomics can reveal novel and unexpected metabolites, while quantitative analysis of specific compounds of interest can be achieved through targeted approaches. For example, three urolithin metabotypes (A, B, and 0) have been defined by metabolomics through the quantification of the production of Urolithin A, Isourolithin A, and Urolithin B in human intervention trials [10].

Metatranscriptomics provides a critical layer of information by measuring which microbial genes are actively expressed in response to a dietary intervention, revealing “what they are doing”. This approach is pivotal in the discovery of a novel polyphenol‐metabolizing enzyme. For example, metatranscriptomics can be used to identify catechol‐dehydroxylase‐encoding genes in response to specific dietary polyphenols. The key strength of this approach is its ability to distinguish between genes that are simply present and those that are activated to promote metabolism under specific conditions.

Bioinformatics plays a crucial integrating role, with sophisticated computational tools employed for the processing of extensive data sets from multi‐omics. The employment of statistical models (e.g., MaAsLin and LEfSe) and network analyses like WGCNA (weighted gene co‐expression network analysis) is instrumental in the generation of powerful association networks. These networks establish connections between specific microbes, their genes and transcripts, and the production of crucial metabolites. Beyond correlation‐based methodologies, bioinformatics has the capacity to utilize chemical structure similarity to predict unidentified microbial metabolic capabilities. Besides, bioinformatic analysis of chemical space can lead to the generation of testable hypotheses about microbe–drug/diet interactions, thus complementing approaches based on microbial taxonomic or gene abundance correlations.

The next stage aims to refine these broad associations into specific candidate mechanisms. It is critical to transition the emphasis from the taxonomic distribution to functional genetic elements. Functional metagenomics is an analytic method that does not require culturing procedures. It achieves this by reassembling environmental DNA into an alternative host, such as *E. coli*, and screening for fragments possessing specific functions. This methodology is effective in identifying gene clusters involved in the degradation of plant cell‐wall polysaccharides. It is reasonable to hypothesize that this will also lead to the identification of new phytochemical‐converting enzymes from uncultured human gut microbes. The key advantage is that functional metagenomics directly links a phenotype to a genetic fragment without requiring prior knowledge of the organisms involved.

The field of comparative genomics has seen a considerable increase in the availability of sequenced bacterial genomes, which has led to significant advancements in research. The comparison of the genomes of metabolic function strains (e.g., S‐equol producers) with those that do not possess this function allows the identification of gene clusters that are found exclusively amongst the former and are thus conserved. For instance, the S‐equol‐producing gene cluster demonstrates a high degree of conservation across species of Eggerthellaceae, as evidenced by Dufault‐Thompson et al. through the utilization of comparative genomics, and evidence was also provided for its horizontal transfer to *L. garvieae* 20‐92 [19].

Genome mining and machine learning approaches use bioinformatic algorithms to scan genomes for gene clusters with specific features predictive of involvement in xenobiotic metabolism. For example, Guthrie et al. have devised a machine learning model that utilizes the chemical landscape of the human gut to predict microbe‐mediated biotransformation of foods and drugs. These computational approaches are of particular value in the identification of candidate enzymes from uncultured organisms represented only by metagenome‐assembled genomes.

Multi‐omics integration approaches have been shown to make significant contributions to the discovery of microbial gene clusters associated with phytochemical metabolism. The identification of the *ucd* operon was not achieved through random screening but rather through targeted metagenomic and metatranscriptomic analyses of samples from individuals with defined urolithin metabotypes, followed by comparative genomics to identify conserved gene clusters in *Enterocloster* species [14, 15]. The *ucd* operon, which encodes a molybdopterin‐dependent enzyme complex, would be difficult to predict in advance, but it emerged as a strong candidate through the analysis of multi‐omic correlations and genomic context. This underscores the hypothesis that systematic application of multi‐omics and bioinformatics can dramatically accelerate the discovery of novel microbial functions and provide well‐defined candidate mechanisms for subsequent biochemical validation.

### The rigor of classic biochemistry for experimental validation

In many cases, the mining process through multi‐omics and bioinformatics is merely a hypothesis or strong correlation. To establish causality, we must turn to the controlled environment of the lab bench for validation. This validation pipeline generally involves target strain isolation and culturing, in vitro biotransformation validation, enzymology and genetics validation, and functional validation.

First, the suspected bacterium strains should be isolated from human fecal samples and their predicted metabolic activity can be confirmed. Although metagenomics can reveal the functional genes, it cannot prove that a specific microbe is responsible for the biotransformation in vivo. Culture‐dependent method is still of paramount importance. Guided by multi‐omic predictions, selective cultivation can be employed to isolate target strains from fecal samples. This may involve enrichment cultures with the phytochemical as a substrate, or the use of specific antibiotics or carbon sources to select for particular species. For example, the isolation of *G. urolithinfaciens* and *E. isourolithinifaciens* was achieved through enrichment cultivation of fecal samples from individuals with defined urolithin metabotypes, using ellagic acid as a key substrate [11, 12]. Once a candidate strain is isolated, its metabolic capability should be confirmed in controlled anaerobic cultures. The pure bacterial strain should be incubated with the pure phytochemical precursor, with analytical techniques like high‐performance liquid chromatography and mass spectrometry used to confirm the target metabolite produced by the bacterium definitely. This step provides the first direct evidence that the strain alone can perform the biotransformation.

When the bioconversion is confirmed, specific microbial enzymes and encoding genes (or gene cluster/operon) should be identified, and genetic knockouts or heterologous expression of the candidate genes in a different host microorganism is preferred to characterize enzyme functions, although in some cases it is challenging for the genetic engineering of anaerobic bacteria and anaerobic enzymes. Identifying specific enzymes responsible for biotransformation typically involves a combination of approaches, including activity‐guided protein purification, comparative genomics, and heterologous expression. Once a candidate gene is identified, it will be cloned into a suitable host (e.g., *E. coli*), where the recombinant protein is expressed and purified. Biochemical assays with the purified enzyme and its proposed substrate are then performed to confirm the predicted catalytic activity and to characterize its kinetics, cofactor requirements, and substrate specificity. These approaches have been used to validate enzymes involved in the S‐equol biosynthesis pathway. For instance, Dong et al. employed these methods to characterize Cber, Cadh, and eCadh from *E. lenta*, providing the molecular‐level insights into flavan‐3‐ol C‐ring cleavage and dihydroxylation [22]. In the native or alternative host, candidate genes are disrupted using genetic tools. If the disruption abolishes the metabolic function, and reintroducing the gene via a plasmid restores it, this provides in vivo evidence for the gene's essential role. This strategy was pivotal in confirming the function of the *ucd* operon in *Enterocloster* species. Pidgeon et al. demonstrated that disruption of the *ucd* operon abolished the production of Urolithin A from Urolithin C, and complementation restored it, providing definitive proof that this operon is critical to the final dehydroxylation step [14].

Finally, the purified metabolite is tested in cell cultures or animal models to confirm and compare its bioactivity (e.g., anti‐inflammatory effect) and elucidate its mechanism of action, confirming its role in health. Prior to transitioning to animal models, it is typically necessary to first confirm that the predicted response occurs within complex human fecal communities. This can be achieved by culturing phytochemical substrates alongside fresh fecal slurry and monitoring metabolite production over time, with establishing appropriate heat‐inactivated controls to account for non‐biological degradation effects. Furthermore, the gold standard for establishing in vivo causality is the use of germ‐free animals colonized with defined microbes. If a particular strain or microbial community can metabolize a specific phytochemical and the resulting metabolite is observed solely within colonized animals, this provides the evidence that the microorganism(s) are capable of achieving biotransformation within the host. Finally, the translational relevance of the identified mechanisms can be assessed by returning to human populations. If a particular enzyme or gene cluster is proposed as the cause of a clinically relevant metabolic phenotype (e.g., S‐equol producer status, or urolithin metabotype A), then its abundance or presence in the population should be associated with that phenotype.

## 3 THE FUTURE OF FUNCTIONAL FOODS: A PERSONALIZED AND FERMENTED HORIZON

Deciphering these fundamental host‐microbe‐diet interactions is not merely an academic exercise. It paves the way for a radical innovation in how we approach nutrition, health, and food production. The future lies in shifting our focus from the dietary precursor to the bioactive microbial metabolite, which is shaped by four key strategies as discussed below.

### Next‐generation probiotics as “specialist converters”

The concept of probiotics has evolved considerably over the past two decades, moving from general wellness supplements and often undefined claims to highly targeted, function‐specific applications. NGPs, also referred to as live biotherapeutic products, are defined as well‐characterized strains derived from the healthy human gut microbiota, and these strains are developed for the prevention or treatment of specific diseases or conditions. Unlike traditional probiotics (e.g., *Lactobacillus* and *Bifidobacterium* species), NGPs are selected based on the mechanism of their metabolic capabilities, their interactions with the host, and their ability to colonize in the gut ecosystem, rendering them ideal “specialized converters” capable of addressing specific metabolic deficiencies within an individual's gut microbiota.

Traditional probiotics offer a range of health benefits, but their mode of action is often multifaceted and strain‐dependent, making it difficult to predict clinical outcomes in specific individuals. By contrast, the development of NGP has a clearly defined mechanism. For example, *E. limosum* has been proposed as an NGP candidate due to its ability to produce the potent phytoestrogen 8‑prenylnaringenin from hops‑derived isoxanthohumol [24]. Because of the defined biochemical pathways, the efficacy of NGPs can be measured using specific metabolic biomarkers (e.g., Urolithin A or S‐equol levels), enabling a precision medicine approach.

The developmental trajectory for such specialized converters may draw upon the case of *Akkermansia muciniphila*. Although not a primary phytochemical converter, this mucin‐degrading bacterium has been associated with improved metabolic health and has undergone rigorous preclinical and clinical evaluation. Notably, *A. muciniphila* has been demonstrated with good safety and preliminary efficacy in overweight and obese humans. This particular case demonstrates that NGPs can be delivered alive, pasteurized, or even as purified membrane proteins based on the mechanism of action.

Despite the potential of these novel genome‐based therapeutics, numerous challenges remain in advancing them from the laboratory stage to clinical application. The first key point is the colonization resistance, which means the ability of an exogenous microbe to survive in the recipient's gut, which is highly variable and depends on many factors such as baseline microbiota, dietary habits, and the intestinal niches. For a specialist converter NGP, long‑term survival may be not necessary, but it should be sufficient to produce the desired metabolite, as suggested by the Urolithin A studies [14]. Nevertheless, strategies to enhance colonization, such as co‑administration with a specific prebiotic or encapsulation technologies, are the focus of active investigation. In addition, the complex ecological networks in human gut, including the example of cross‑feeding interactions between *A. muciniphila* and butyrate‑producing bacteria such as *Anaerostipes caccae* and *Anaerobutyricum hallii*, indicate that successful colonization may depend on the broader microbial community.

Safety is also important because many NGP candidates may belong to genera that contain both commensal and pathogenic members (e.g., *Eggerthella*, *Enterocloster*, and *Eubacterium*). Thus, an NGP candidate must be conducted a comprehensive safety assessment before application, including genome sequencing to rule out virulence factors or antibiotic resistance genes, and toxicological studies in animal models. For instance, the successful progression of *A. muciniphila* through clinical trials demonstrates that such rigorous preclinical evaluation can pave the way for regulatory approval, even for strains not traditionally used as probiotics.

Individual tolerance and host–microbe interactions also require careful consideration. The efficacy of an NGP may differ between individuals due to genetic background, immune status, and intrinsic gut microbiota. Some individuals may exhibit an inflammatory response to a newly introduced strain, while others may tolerate it well, which underscores the need for careful dose‑escalation studies and the inclusion of diverse populations in early‑phase trials. The use of defined microbial consortia rather than single strains, as demonstrated in the successful treatment of recurrent *Clostridium difficile* infection with a multi‑species cocktail, may reduce the risk of excessive immune reactions by mimicking the natural complexity of the microbiota.

### Personalized synbiotics and precision prebiotics

Although NGPs can provide missing metabolic functions, their successful translation still faces practical challenges, including unstable colonization rates, host tolerance, and the requirement for sustained metabolic activity. Personalized synbiotics (defined as combinations of specific NGP and their preferred substrates) and precision prebiotics (dietary compounds designed to enrich specific microorganisms with desirable metabolic capabilities) offer complementary strategies to overcome the identified challenges. Crucially, both approaches can be designed according to an individual's baseline microbiota and metabotype, thereby achieving the truly personalized nutrition.

The development of synbiotic formulations is primarily based on two key considerations. Co‐administration of substrates confers a competitive advantage to introduced strains, enhancing their opportunity to achieve transient colonization and metabolic activity without requiring permanent establishment, simultaneously inducing the expression of genes required for the biotransformation process, thereby maximizing metabolic output. For example, a synbiotic formulation designed for Urolithin A non‑producers may combine bacterial strains of *Gordonibacter* and *Enterocloster* strains with extracts rich in ellagitannins. Preclinical evidence demonstrating the colonization of *Enterocloster* and production of Urolithin A in animals lacking ellagitannin conversion capacity suggests that such a synbiotic may prove effective even without long‑term colonization [14, 28]. Similarly, an S‐equol‑producing synbiotic might combine *S. isoflavoniconvertens* and soy isoflavones, enabling the substrate to be effectively utilized at the appropriate time and location where the NGP is active.

For individuals whose essential microbial functions are inherently at a lower level, precision prebiotics may offer a simpler and more cost‐effective alternative to live bacteria supplements. The goal is to identify dietary compounds that specifically stimulate the growth and activity of the target microbe(s) without influencing the microbial community. For instance, certain polyphenols (e.g., cranberry and grape polyphenols) have been shown to increase the abundance of *A. muciniphila* in mice and humans, which is likely achieved by creating a favorable gut environment or acting as an indirect substrate. Mechanistically, polyphenols may enhance mucus thickness, thereby providing more substrate for mucin‑degrading specialists like *Akkermansia*. Within the context of phytochemical biotransformation, precisely engineered prebiotics can be designed to enrich indigenous *Gordonibacter* or *Enterocloster* populations in individuals who possess these microbes but exhibit suboptimal functional levels. This first requires determining the ecological niche and trophic preferences of the target taxonomic group.

To translate concepts into clinical applications, multiple types of research must be conducted. Microbiological ecology studies should elucidate both the nutritional preferences of key translational strains and their ecological interactions. For example, by identifying which polyphenols specifically promote the growth of *Gordonibacter* or *Enterocloster* without stimulating pathogens proliferation. Then, proof‑of‑principle trials of synbiotics in metabotype‑stratified individuals should compare the effects of NGP monotherapy, substrate monotherapy, and the synbiotics combination therapy. It should also precisely measure target metabolite levels in plasma and urine, while assessing safety, tolerability, and the duration of effect following discontinuation. Furthermore, longitudinal observational studies are required to track the stability of metabolic types and their relationship with diet, antibiotic use, and other lifestyle factors, thereby enabling more accurate prediction of which populations may benefit from specific interventions. Finally, employing germ‐free mice colonized with synthetic microbiota designed to mimic human metabolic profiles can elucidate the effects of specific prebiotics on microbial community structure and metabolic outputs.

### Postbiotics: Innovation of the gut metabolites as nutraceuticals

The most direct way to overcome interindividual variability is to bypass microbial conversion altogether, which can be achieved through “postbiotics”, the administration of the final, purified bioactive metabolites of phytochemical precursors. By identifying, producing (often through industrial fermentation), and purifying compounds like Urolithin A or S‐equol, we can deliver a standardized, pharmacologically precise dose. This ensures a reliable physiological response for everyone, regardless of their gut microbiota composition.

The identification of postbiotics needs to elucidate the gut microbial metabolic pathway. For instance, by comparing metabolomics and activity‐directed fractionation techniques, and combining in vitro and in vivo validation, it has been recognized that it is not the parent ellagitannins but Urolithin A that mediates the mitochondrial health benefits [9]. Once a metabolite is identified as the key bioactive substance, its production must be scalable. This can be achieved through industrial fermentation using specific microbial strains or their consortium, followed by purification to meet pharmaceutical‐grade standards. The case of Urolithin A exemplifies this advancement. Following its identification as the primary intestinal metabolite of ellagitannins, researchers developed a scalable production process enabling its preclinical and clinical evaluation [17].

A robust postbiotic development pathway requires rigorous preclinical safety assessment. Such studies are crucial for meeting regulatory requirements and establishing the safe dose for human trials. Subsequent Phase I clinical trials in healthy volunteers will assess the drug's safety, tolerability, and pharmacokinetic properties. For example, a randomized, double‐blind, placebo‐controlled trial demonstrated that daily administration of 1 gram of Urolithin A for 4 months was safe and well tolerated [9]. Subsequent Phase II clinical trials conducted in middle‐aged and elderly populations demonstrated improvements in participants' muscular endurance, mitochondrial biomarkers, and physical function [18]. This sequence, including mechanistic discovery, preclinical safety, first‐in‐human and proof‐of‐concept efficacy, provides a template for other postbiotic candidates, such as S‐equol. Although there is already a market for S‐equol as a supplement in some countries, its clinical evidence is actually insufficient.

The successful activation of Urolithin A has stimulated research interest in developing other gut metabolites as postbiotics, including S‑equol, 8‑prenylnaringenin, and phenyl‑γ‑valerolactones. For each, the same principles must be followed by clearly identifying the bioactive components, achieving scalable production, conducting rigorous preclinical safety assessments, designing clinical trials to evaluate both efficacy and safety, and monitoring gut microbiota simultaneously. However, there are still some challenges, such as the need for long‑term safety data and the development of formulations that enhance oral bioavailability. Furthermore, this field requires investigation into whether postbiotic combinations, such as Urolithin A and S‐equol, can yield additive or synergistic benefits, thereby mimicking the natural diversity of microbial metabolites produced in complex diets.

### Food fermentation 2.0: Precision fermentation

The strategies discussed above—NGPs, synbiotics, and postbiotics—all aim to deliver specific microbial functions or their metabolic products to the human host. Another complementary approach involves utilizing these microbial metabolic capabilities in vitro to pre‐transform phytochemicals within a food matrix before consumption. This concept, often termed precision fermentation, integrates traditional food fermentation techniques with cutting‐edge synthetic biology and process engineering technologies. Unlike the use of probiotics (which deliver live microbes to the gut) or postbiotics (which supply purified metabolites), precision fermentation uses microorganisms as “cell factories” to produce target compounds within controlled industrial environments, yielding components suitable for formulation in functional foods, beverages, or dietary supplements. This approach offers several advantages that it eliminates the need to account for inter‐individual variations in gut microbial metabolism, allows for precise control over product composition, and enables large‐scale production to meet market demand.

The selection of microorganisms for precision fermentation builds directly upon the mechanistic knowledge of NGPs and postbiotics. Rather than simply using gut‐derived specialist converters, a more practical approach is to use them in conjunction with traditional fermentation strains to create functional foods with enhanced health benefits, as NGPs may not grow well outside the gut. For example, one could envision adding daidzein and an S‐equol‑producing NGP such as *S. isoflavoniconvertens* to a standard yoghurt starter culture (*Lactobacillus bulgaricus* and *Streptococcus thermophilus*), thereby producing a fermented dairy product enriched with S‑equol. This type of concept raises several practical challenges that must be addressed. First, gut‑derived anaerobes like *Slackia* or *Gordonibacter* are poorly adapted to the aerobic conditions and acidic environment of food fermentation; however, the oxygen‑consuming activity of the traditional starter cultures may progressively establish a locally anaerobic microenvironment during fermentation, thereby enabling the NGPs to survive and complete their biotransformation. Second, the safety of introducing these gut bacteria into food products must be rigorously evaluated, including genome sequencing to rule out virulence factors or antibiotic resistance genes, as discussed before. Third, consistent production of desired metabolites (e.g., S‐equol and Urolithin A) requires that the NGPs remain metabolically active during fermentation.

An alternative strategy is to circumvent these challenges by transferring the key biosynthetic genes, such as the S‐equol pathway enzymes or urolithin dehydroxylases, into well‑characterized, GRAS‑status industrial hosts like *Saccharomyces cerevisiae* or *E. coli*. These strains have been optimized for large‐scale fermentation and can be genetically engineered to achieve high yields of target compounds. This gene transfer approach has been successfully demonstrated for S‐equol production in *L. lactis* and for Urolithin A enzymes in *E. coli*, paving the way for scalable production of these bioactive metabolites.

Beyond the strategies of co‑cultivation with traditional starters and genetic engineering, another powerful approach is adaptive laboratory evolution (ALE). ALE induces spontaneous mutation in probiotics to enrich for spontaneous mutants with improved tolerance and conversion efficiency under specific conditions, such as exposure to the target phytochemical, low pH, or conditions mimicking the food matrix. This method requires no prior genetic knowledge and can yield robust strains that are already adapted to the intended production environment. For instance, lactic acid bacteria used in dairy fermentations have been evolved to better tolerate oxidative stress and acid, and these traits will benefit any gut‑derived NGP co‑cultured in yoghurt or cheese. ALE offers a complementary, non‑transgenic route to enhance the performance of both traditional fermentation organisms and NGPs within complex food matrices, improving their survival and metabolic output without direct genetic modification.

Precision fermentation is not intended to replace nutritional supplements, synbiotics or postbiotics, but rather to serve as a complementary approach to broaden the spectrum of functional foods. For instance, a fermented beverage containing pre‐converted Urolithin A could function as a postbiotic‐like product, while yoghurt fermented using strains producing S‐equol would constitute a form of synbiotic. The choice of approach depends on factors such as cost, consumer acceptance, regulatory pathways, and the speed at which desired outcomes can be achieved. Synthetic biology, including CRISPR‐based genome editing, extracellular systems, and machine learning‐driven pathway optimization, will continue to accelerate the development of precision fermentation processes. Moreover, as our understanding of gut microbial metabolic mechanisms deepens, the range of compounds that can be targeted will continue to expand, enabling the development of a new generation of fermented foods designed to deliver consistent, science‐backed health benefits.

## 4 CONCLUSION

The field of nutrition is at a pivotal crossroad. The simple model of direct absorption is giving way to a more nuanced and exciting reality where our gut microbiota stands as the critical mediator between diet and health. The inconsistency of phytochemical efficacy in humans is not a dead end but a doorway to a deeper understanding of our profound symbiotic relationship with our gut microbes. By integrating multi‐omics, bioinformatics, and classic biochemistry, we are finally beginning to map the microbial metabolic mystery that underpins the health benefits of our diet. The path forward is clear that we must invest in this fundamental research to move from a few well‐understood examples to a comprehensive atlas of phytochemical metabolism in our gut. In doing so, we will not only solve the efficacy paradox but also lay the foundation for a new era of personalized nutrition. We will be able to precisely tailor diets, supplements, and functional foods to our unique microbial makeup, finally unlocking the profound and long‐promised health potential hidden within our food. The future of functional ingredients lies not merely in the plant itself, but more importantly in the potent molecules created by our gut microbiome.

## AUTHOR CONTRIBUTIONS

**Peng Yang**: Writing—original draft; visualization. **Ren‐You Gan**: Conceptualization; writing—original draft; writing—review and editing; funding. All authors have read the final manuscript and approved it for publication.

## CONFLICT OF INTEREST STATEMENT

Ren‐You Gan holds the position of Executive Editor for iMetaOmics, and the authors declare no other conflicts of interest.

## ETHICS STATEMENT

No animals or humans were involved in this study.

## AI STATAMENT

During the preparation of this work, we used DeepSeek and Gemini 3 to refine and improve the proposed ideas, as well as to check grammar and sentence coherence. After using these tools, we reviewed and edited the content as needed.

## Data Availability

Supplementary materials (graphical abstract, slides, videos, Chinese translated version, and updated materials) may be found in the online DOI or iMetaOmics http://www.imeta.science/imetaomics/.

## References

[1] Zhang, Yu‐Jie , Ren‐You Gan , Sha Li , Yue Zhou , An‐Na Li , Dong‐Ping Xu , and Hua‐Bin Li . 2015. “Antioxidant Phytochemicals for the Prevention and Treatment of Chronic Diseases.” Molecules 20: 21138–21156. 10.3390/molecules201219753 PMC6331972 26633317

[2] Ashrafpour, Sahand , and Manouchehr Ashrafpour . 2025. “The Double‐Edged Sword of Nutraceuticals: Comprehensive Review of Protective Agents and Their Hidden Risks.” Frontiers in Nutrition 12: 2025. 10.3389/fnut.2025.1524627 PMC11984464 40212723

[3] Magee, Pamela J . 2011. “Is Equol Production Beneficial to Health?” Proceedings of the Nutrition Society 70: 10–18. 10.1017/S0029665110003940 21092366

[4] Mayo, Baltasar , Lucía Vázquez , and Ana Belén Flórez . 2019. “Equol: A Bacterial Metabolite From the Daidzein Isoflavone and Its Presumed Beneficial Health Effects.” Nutrients 11: 2231. 10.3390/nu11092231 PMC6770660 31527435

[5] Zhernakova, Alexandra , Alexander Kurilshikov , Marc Jan Bonder , Ettje F. Tigchelaar , Melanie Schirmer , Tommi Vatanen , Zlatan Mujagic , et al. 2016. “Population‐Based Metagenomics Analysis Reveals Markers for Gut Microbiome Composition and Diversity.” Science 352: 565–569. 10.1126/science.aad3369 PMC5240844 27126040

[6] Jia, Baolei , Xiao Han , Kyung Hyun Kim , and Che Ok Jeon . 2022. “Discovery and Mining of Enzymes From the Human Gut Microbiome.” Trends in Biotechnology 40: 240–254. 10.1016/j.tibtech.2021.06.008 34304905

[7] Noerman, Stefania , Marjukka Kolehmainen , and Kati Hanhineva . 2020. “Profiling of Endogenous and Gut Microbial Metabolites to Indicate Metabotype‐Specific Dietary Responses: A Systematic Review.” Advances in Nutrition 11: 1237–1254. 10.1093/advances/nmaa031 PMC7490160 32271864

[8] Gandhi, Gopalsamy Rajiv , Poovathumkal James Antony , Stanislaus Antony Ceasar , Alan Bruno Silva Vasconcelos , Monalisa Martins Montalvão , Mariana Nobre Farias de Franca , Ayane de Sá Resende , et al. 2024. “Health Functions and Related Molecular Mechanisms of Ellagitannin‐Derived Urolithins.” Critical Reviews in Food Science and Nutrition 64: 280–310. 10.1080/10408398.2022.2106179 35959701

[9] Andreux, Pénélope A. , William Blanco‐Bose , Dongryeol Ryu , Frédéric Burdet , Mark Ibberson , Patrick Aebischer , Johan Auwerx , Anurag Singh , and Chris Rinsch . 2019. “The Mitophagy Activator Urolithin A Is Safe and Induces a Molecular Signature of Improved Mitochondrial and Cellular Health in Humans.” Nature Metabolism 1: 595–603. 10.1038/s42255-019-0073-4 32694802

[10] Romo‐Vaquero, María , Adrián Cortés‐Martín , Viviana Loria‐Kohen , Ana Ramírez‐de‐Molina , IIzaskun García‐Mantrana , María Carmen Collado , Juan Carlos Espín , María Victoria Selma . 2019. “Deciphering the Human Gut Microbiome of Urolithin Metabotypes: Association with Enterotypes and Potential Cardiometabolic Health Implications.” Molecular Nutrition & Food Research 63: e1800958. 10.1002/mnfr.201800958 30471188

[11] Selma, María V. , David Beltrán , Rocío García‐Villalba , Juan C. Espín , Francisco A. Tomás‐Barberán . 2014. “Description of Urolithin Production Capacity From Ellagic Acid of Two Human Intestinal Gordonibacter Species.” Food & Function 5: 1779–1784. 10.1039/C4FO00092G 24909569

[12] Selma, María V. , David Beltrán , María C. Luna , María Romo‐Vaquero , Rocío García‐Villalba , Alex Mira , Juan C. Espín , and Francisco A. Tomás‐Barberán . 2017. “Isolation of Human Intestinal Bacteria Capable of Producing the Bioactive Metabolite Isourolithin A From Ellagic Acid.” Frontiers in Microbiology 8: 2017. 10.3389/fmicb.2017.01521 PMC5545574 28824607

[13] Iglesias‐Aguirre, Carlos E. , Rocío García‐Villalba , David Beltrán , María Dolores Frutos‐Lisón , Juan C. Espín , Francisco A. Tomás‐Barberán , and María V. Selma . 2023. “Gut Bacteria Involved in Ellagic Acid Metabolism to Yield Human Urolithin Metabotypes Revealed.” Journal of Agricultural and Food Chemistry 71: 4029–4035. 10.1021/acs.jafc.2c08889 PMC9999415 36840624

[14] Pidgeon, Reilly , Sacha Mitchell , Michael Shamash , Layan Suleiman , Lharbi Dridi , Corinne F. Maurice , and Bastien Castagner . 2025. “Diet‐Derived Urolithin A Is Produced by a Dehydroxylase Encoded by Human Gut Enterocloster Species.” Nature Communications 16: 999. 10.1038/s41467-025-56266-2 PMC11760930 39856097

[15] Bae, Minwoo , Xueyang Dong , Julian Avila‐Pacheco , Quyen D. Nguyen , Fechi Inyama , Vayu Hill‐Maini , Clary B. Clish , and Emily P. Balskus . 2025. “Distinct Classes of Gut Bacterial Molybdenum‐Dependent Enzymes Produce Urolithins.” Proceedings of the National Academy of Sciences 122: e2501312122. 10.1073/pnas.2501312122 PMC12771579 41439715

[16] Seyed Hameed, Ahkam Saddam , Parkash Singh Rawat , Xiangfeng Meng , and Weifeng Liu . 2020. “Biotransformation of Dietary Phytoestrogens by Gut Microbes: A Review on Bidirectional Interaction Between Phytoestrogen Metabolism and Gut Microbiota.” Biotechnology Advances 43: 107576. 10.1016/j.biotechadv.2020.107576 32531317

[17] Liu, Sophia , Davide D'Amico , Eric Shankland , Saakshi Bhayana , Jose M. Garcia , Patrick Aebischer , Chris Rinsch , Anurag Singh , and David J. Marcinek . 2022. “Effect of Urolithin A Supplementation on Muscle Endurance and Mitochondrial Health in Older Adults: A Randomized Clinical Trial.” JAMA Network Open 5: e2144279. 10.1001/jamanetworkopen.2021.44279 PMC8777576 35050355

[18] Sekikawa, Akira , Ashley Weaver , Kelly Mroz , Nina Z. Heilmann , Diana A. Madrid Fuentes , Kristen J. Koltun , Lauren J. Carlson , et al. 2025. “S‐Equol as a Gut‐Derived Phytoestrogen Targeting Estrogen Receptor β: A Promising Bioactive Nutrient for Bone Health in Aging Women and Men: A Narrative Review.” Nutrients 17: 3962. doi: 10.3390/nu17243962 https://www.mdpi.com/2072-6643/17/24/3962 10.3390/nu17243962 PMC12735577 41470907

[19] Dufault‐Thompson, Keith , Brantley Hall , and Xiaofang Jiang . 2022. “Taxonomic Distribution and Evolutionary Analysis of the Equol Biosynthesis Gene Cluster.” BMC Genomics 23: 182. 10.1186/s12864-022-08426-7 PMC8898433 35247986

[20] Liu, Chen , Ren‐You Gan , Daiwen Chen , Liang Zheng , Siew Bee Ng , and Ivonne M. C. M. Rietjens . 2024. “Gut Microbiota‐Mediated Metabolism of Green Tea Catechins and the Biological Consequences: An Updated Review.” Critical Reviews in Food Science and Nutrition 64: 7067–7084. 10.1080/10408398.2023.2180478 38975869

[21] Zhang, Shuling , Bingyong Mao , Shumao Cui , Qiuxiang Zhang , Jianxin Zhao , Xin Tang , and Wei Chen . 2024. “Absorption, Metabolism, Bioactivity, and Biotransformation of Epigallocatechin Gallate.” Critical Reviews in Food Science and Nutrition 64: 6546–6566. 10.1080/10408398.2023.2170972 36728926

[22] Dong, Xueyang , Minwoo Bae , Chi Le , Miguel A. Aguilar Ramos , and Emily P. Balskus . 2025. “Enantiocomplementary Gut Bacterial Enzymes Metabolize Dietary Polyphenols.” Journal of the American Chemical Society 147: 7231–7244. 10.1021/jacs.4c09892 PMC11887054 39993729

[23] Lessard‐Lord, Jacob , Charlène Roussel , Valérie Guay , and Yves Desjardins . 2023. “Characterization of the Interindividual Variability Associated with the Microbial Metabolism of (−)‐Epicatechin.” Journal of Agricultural and Food Chemistry 71: 13814–13827. 10.1021/acs.jafc.3c05491 PMC10516121 37683128

[24] Possemiers, Sam , Sylvie Rabot , Juan Carlos Espín , Aurélia Bruneau , Catherine Philippe , Antonio González‐Sarrías , Arne Heyerick , et al. 2008. “Eubacterium limosum Activates Isoxanthohumol from Hops (Humulus lupulus L.) into the Potent Phytoestrogen 8‐Prenylnaringenin In Vitro and in Rat Intestine3.” The Journal of Nutrition 138: 1310–1316. 10.1093/jn/138.7.1310 18567753

[25] Liou, Catherine S. , Shannon J. Sirk , Camil A. C. Diaz , Andrew P. Klein , Curt R. Fischer , Steven K. Higginbottom , Amir Erez , et al. 2020. “A Metabolic Pathway for Activation of Dietary Glucosinolates by a Human Gut Symbiont.” Cell 180: 717–728.e19. 10.1016/j.cell.2020.01.023 PMC7515767 32084341

[26] Bae, Minwoo , Chi Le , Raaj S. Mehta , Xueyang Dong , Lindsey M. Pieper , Lorenzo Ramirez , and Margaret Alexander , et al. 2024. “Metatranscriptomics‐Guided Discovery and Characterization of a Polyphenol‐Metabolizing Gut Microbial Enzyme.” Cell Host & Microbe 32: 1887–1896.e1888. 10.1016/j.chom.2024.10.002 PMC11585353 39471822

[27] Maini Rekdal, Vayu , Elizabeth N. Bess , Jordan E. Bisanz , Peter J. Turnbaugh , and Emily P. Balskus . 2019. “Discovery and Inhibition of an Interspecies Gut Bacterial Pathway for Levodopa Metabolism.” Science 364: eaau6323. 10.1126/science.aau6323 PMC7745125 31196984

[28] Haiser, Henry J. , David B. Gootenberg , Kelly Chatman , Gopal Sirasani , Emily P. Balskus , and Peter J. Turnbaugh . 2013. “Predicting and Manipulating Cardiac Drug Inactivation by the Human Gut Bacterium Eggerthella lenta.” Science 341: 295–298. 10.1126/science.1235872 PMC3736355 23869020

